# Absorbance measurements of oxidation of homogentisic acid accelerated by the addition of alkaline solution with sodium hypochlorite pentahydrate

**DOI:** 10.1038/s41598-018-29769-w

**Published:** 2018-07-27

**Authors:** Yasunori Tokuhara, Kenichi Shukuya, Masami Tanaka, Keisuke Sogabe, Yasukazu Ejima, Sho Hosokawa, Hiroyuki Ohsaki, Tatsuya Morinishi, Eiichiro Hirakawa, Yutaka Yatomi, Tatsuo Shimosawa

**Affiliations:** 1 Department of Medical Technology, Ehime Prefectural University of Health Sciences, Ehime, Japan; 2 Department of Clinical Laboratory, School of Medicine, the University of Tokyo, Tokyo, Japan; 3 Kaneka Techno Research Corporation, Hyogo, Japan; 4 Kaneka Corporation, Vinyls and Chlor-Alkali Solutions Vehicle, Osaka, Japan; 5 Department of Medical Biophysics, Kobe University Graduate School of Health Sciences, Kobe, Japan; 6 Department of Medical Technology, Kagawa Prefectural University of Health Sciences, Kagawa, Japan; 7 Clinical Laboratory Medicine, School of Medicine, International University of Health and Welfare, Chiba, Japan

## Abstract

The urine of patients with alkaptonuria turns dark brown due to the oxidation of homogentisic acid (HGA) to benzoquinone acetic acid (BQA), and this is accelerated by the addition of alkali. We recently reported that alkaptonuric urine and HGA after the addition of alkali showed characteristic peaks at 406 and 430 nm. In order to improve the sensitivity of our spectrometric method for the detection of HGA, we accelerated the oxidation of HGA to BQA using sodium hypochlorite pentahydrate (NaOCl·5H_2_O), which is a strong oxidant. In the present study, we measured the absorption spectra of alkaptonuric urine and HGA solution after the addition of sodium hydroxide (NaOH) or NaOH with NaOCl·5H_2_O and analyzed the oxidation reaction of HGA after alkalization using a liquid chromatography time-of-flight mass spectrometer (LC/TOF-MS) and nuclear magnetic resonance (NMR) spectrometry. We accelerated the oxidation of HGA to BQA by adding NaOH with NaOCl·5H_2_O, and this absorbance measurement was useful for more sensitively observing the oxidation of HGA than LC/TOF-MS and NMR spectroscopy. This quick and easy screening method may be suitable for the diagnosis of alkaptonuria.

## Introduction

Alkaptonuria, a hereditary metabolic disorder, occurs due to the absence of the enzyme homogentisic acid (HGA) oxidase. This defect leads to the accumulation of HGA, an intermediate in the catabolism of phenylalanine and tyrosine^1,2^. The urine of patients with alkaptonuria turns dark brown at room temperature after several hours to days; the oxidation of HGA to benzoquinone acetic acid (BQA) underlies this color change, which is accelerated by the addition of alkali^3,4^. The accumulation of HGA and its metabolites in tissues causes ochronosis, which is characterized by the darkening of cartilaginous tissues and bone, and may lead to arthritis, joint destruction, and the deterioration of cardiac valves^5–7^.

In recent years, the measurement of HGA in urine has become possible using gas chromatography-mass spectrometry, a high-performance liquid-chromatographic (HPLC) method, and nuclear magnetic resonance (NMR) spectrometry^8,9^. However, these methods are very expensive and difficulties are associated with manipulating and maintaining machines. In an attempt to develop a quick and easy screening test for alkaptonuria using a spectrophotometer, we recently reported novel visible-light region absorbance peaks in the urine of patients with alkaptonuria after alkalization^10^. Alkaptonuric urine and HGA solution exhibit characteristic absorption spectra with peaks at 406 and 430 nm that appear one minute after alkalization. This method enables quick and easy screening to detect the oxidation of HGA to BQA in urine.

In the present study, in an attempt to improve the sensitivity of our spectrometric method for the detection of HGA, we accelerated the oxidation of HGA to BQA using sodium hypochlorite pentahydrate (NaOCl·5H_2_O), a strong oxidant with a solid (finely ground) form and an effective chlorine concentration of approximately 42%. Moreover, using a liquid chromatography time-of-flight mass spectrometer (LC/TOF-MS) and NMR spectrometry, we analyzed the oxidation reaction of HGA to BQA after alkalization and examined how interference substances affect the oxidation of HGA. The results obtained suggest that the addition of alkaline solution with NaOCl·5H_2_O to alkaptonuric urine accelerates the oxidation of HGA to BQA, and the measurement of the absorption spectrum in the visible region is useful for observing a urine color change due to the oxidation of HGA to BQA.

## Results

### Color change and absorption spectra in the visible region

**Figure 1:**
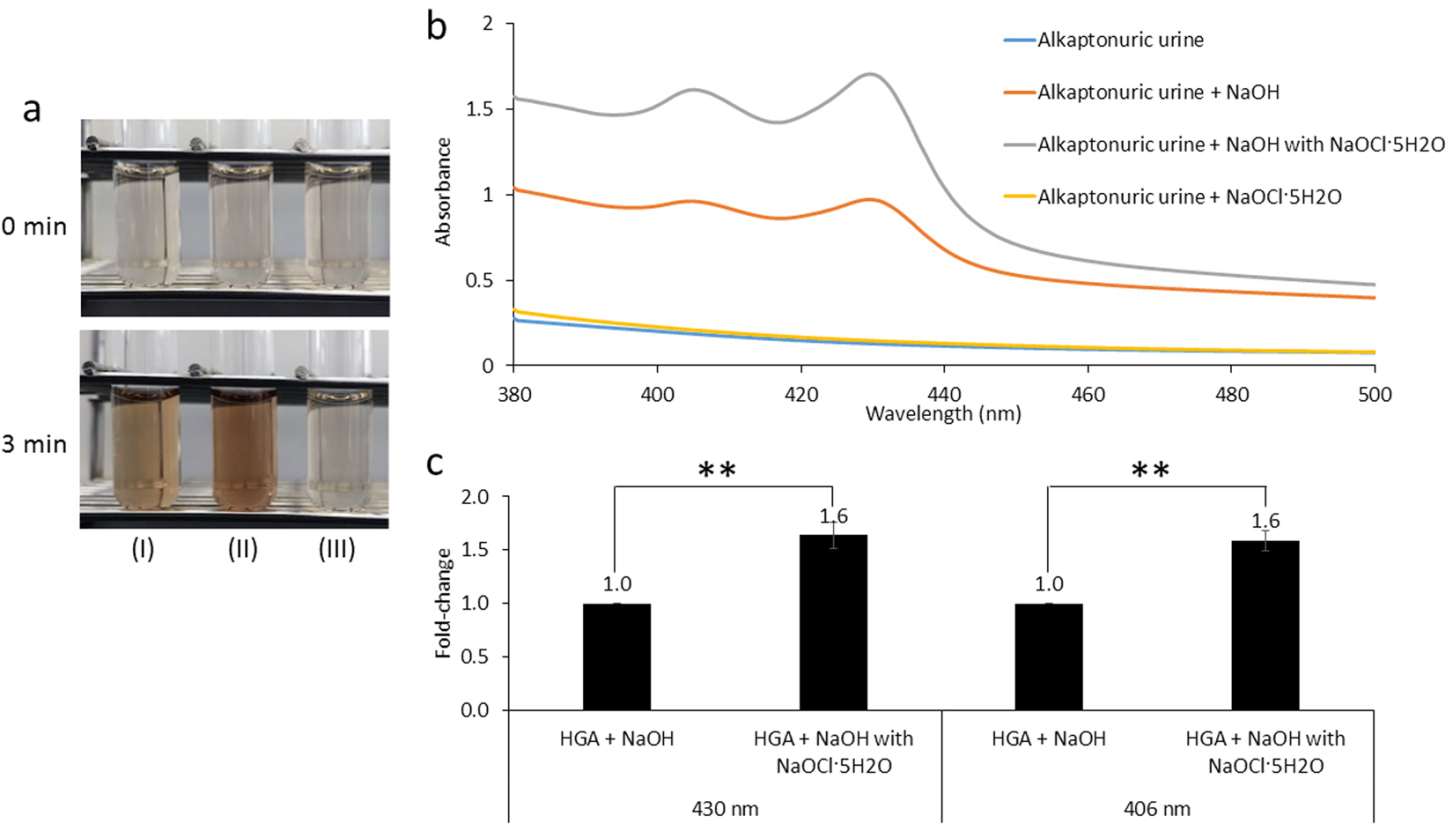
Color change and absorption spectra of alkaptonuric urine. (**a**) Alkaptonuric urine (HGA concentration of 271 mg/L) after the addition of NaOH (I), NaOH with NaOCl·5H_2_O (II), or NaOCl·5H_2_O (III). (**b**) Absorption spectra of alkaptonuric urine after the addition of NaOH, NaOH with NaOCl·5H_2_O, or NaOCl·5H_2_O. (**c**) Absorbance values at 430 and 406 nm of alkaptonuric urine after the treatment with NaOH or NaOH with NaOCl·5H_2_O. Data are the mean ± SD (n = 3). **P < 0.01.

**Figure 2:**
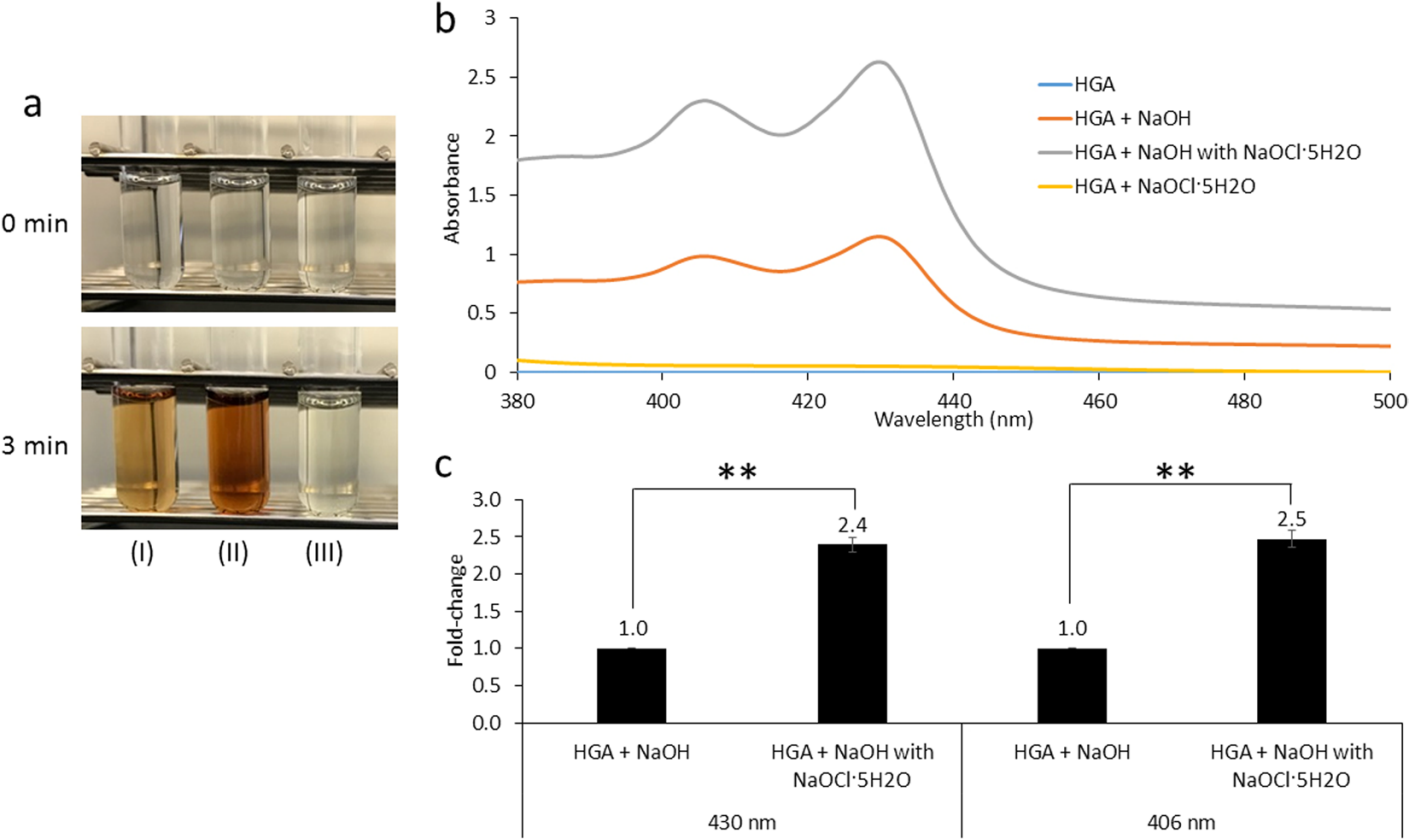
Color change and absorption spectra of 800 mg/L HGA. (**a**) 800 mg/L HGA after the addition of NaOH (I), NaOH with NaOCl·5H_2_O (II), or NaOCl·5H_2_O (III). (**b**) Absorption spectra of 800 mg/L HGA after the addition of NaOH, NaOH with NaOCl·5H_2_O, or NaOCl·5H_2_O. (**c**) Absorbance values at 430 and 406 nm of 800 mg/L HGA after the addition of NaOH or NaOH with NaOCl·5H_2_O. Data are the mean ± SD (n = 3). **P < 0.01.

We initially added NaOH, NaOH with NaOCl·5H_2_O, or NaOCl·5H_2_O to the urine sample collected from the alkaptonuria patient and observed changes in its color. Urine became a darker brown following the addition of NaOH with NaOCl·5H_2_O than with the addition of NaOH (Fig. 1a). However, when the urine sample was incubated with NaOCl·5H_2_O, it did not show a color change (Fig. 1a). We then conducted a spectrophotometric analysis in the visible region (380–500 nm) to detect the absorption curve of the urine sample. The absorbance curve of the urine sample showed sharper peaks following the addition of NaOH with NaOCl·5H_2_O than with the addition of NaOH (Fig. 1b). Furthermore, the absorbance values at 406 and 430 nm of the urine sample were significantly higher following the addition of NaOH with NaOCl·5H_2_O than with the addition of NaOH (Fig. 1c). Similar results were obtained for the HGA solution (Fig. 2).

### Absorption spectra in the UV region

**Figure 3:**
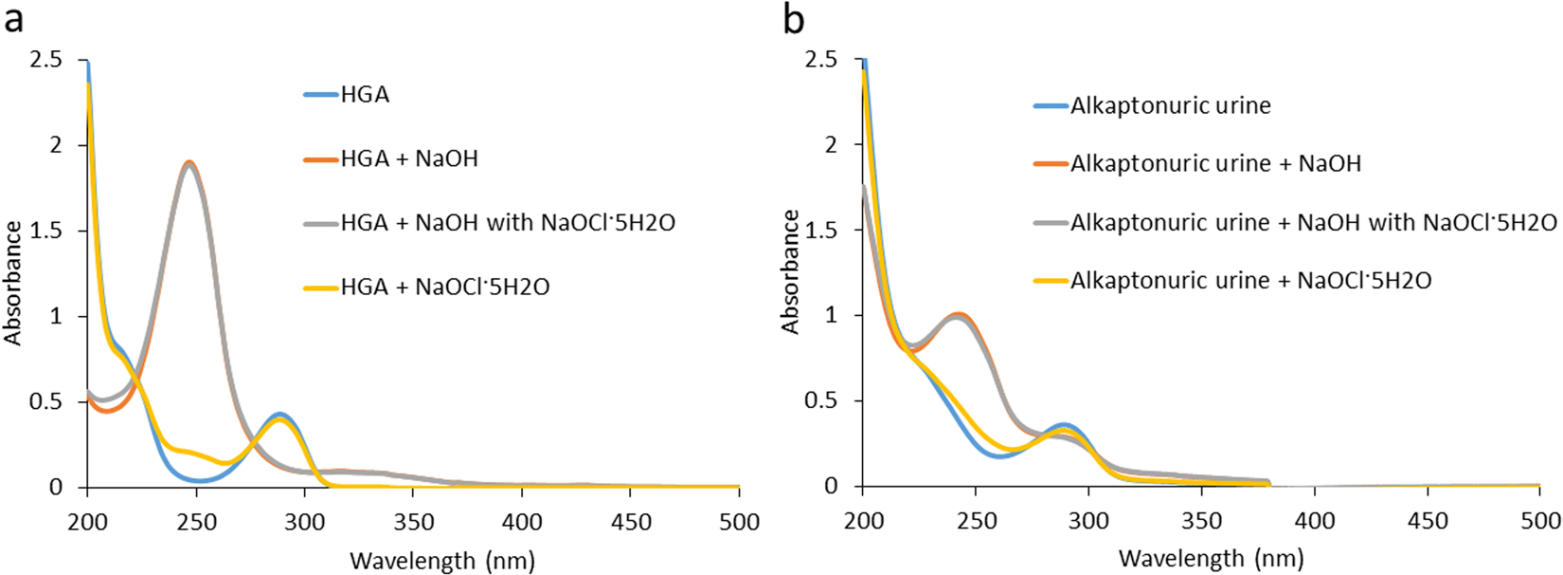
Absorption spectra at 200–500 nm covering the UV region. (**a**) Absorption spectra of 800 mg/L HGA after the addition of NaOH, NaOH with NaOCl·5H_2_O, or NaOCl·5H_2_O at 200–500 nm covering the UV region. (**b**) Absorption spectra of alkaptonuric urine (HGA concentration of 271 mg/L) after the addition of NaOH, NaOH with NaOCl·5H_2_O, or NaOCl·5H_2_O at 200–500 nm covering the UV region.

We then examined absorption spectra at 200–500 nm covering the UV region obtained following the addition of NaOH with NaOCl·5H_2_O to HGA solution. The shift in the absorbance peak from 290 to 250 nm after the addition of NaOH with NaOCl·5H_2_O to HGA solution was identical to that following the addition of NaOH (Fig. 3a). However, a significant difference was not observed between the absorbance values at 250 nm after the addition of NaOH with NaOCl·5H_2_O and after that of NaOH (Fig. 3a). Two characteristic peaks at 406 and 430 nm of HGA after the addition of NaOH or NaOH with NaOCl·5H_2_O disappeared at the time of the measurement in the UV region (Figs 2b and 3a). Similar changes in absorbance peaks were observed for the urine of the patient with alkaptonuria (Fig. 3b).

### Absorption spectra of different concentrations of HGA solutions

**Figure 4:**
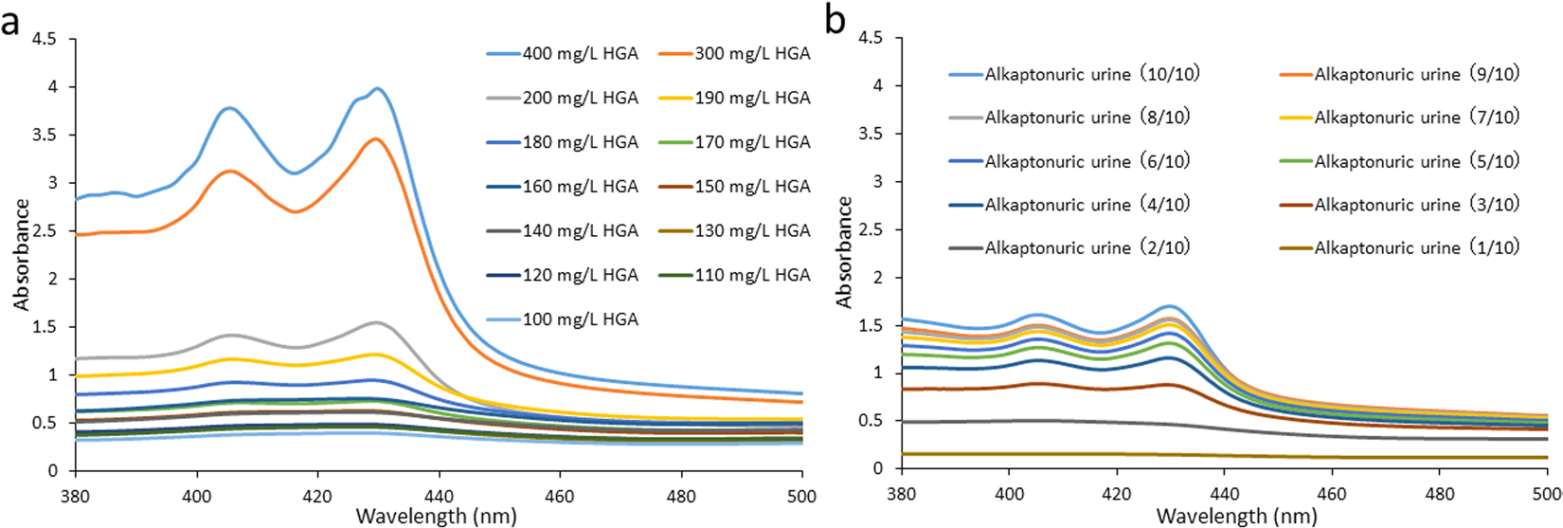
Absorption spectra of HGA and alkaptonuric urine. (**a**) Absorption spectra of HGA solutions (400, 300, 200, 190, 180, 170, 160, 150, 140, 130, 120, 110, and 100 mg/L) after the addition of NaOH with NaOCl·5H_2_O. (**b**) Absorption spectra of alkaptonuric urine (HGA concentration of 271 mg/L) diluted with distilled water (10/10, 9/10, 8/10, 7/10, 6/10, 5/10, 4/10, 3/10, 2/10, and 1/10 dilution) after the addition of NaOH with NaOCl·5H_2_O.

The sample solution of 800 mg/L HGA was diluted sequentially with distilled water and three different HGA solutions (from 400 to 100 mg/L) were prepared. These HGA solutions were incubated with NaOH with NaOCl·5H_2_O, and their absorption spectra were measured (Fig. 4a). The sample solutions of 400, 300, 200, 190, and 180 mg/L HGA showed peaks at 406 and 430 nm, whereas 170 mg/L HGA showed no peaks (Fig. 4a). The two peaks at 406 and 430 nm decreased as HGA solutions were diluted. Alkaptonuric urine, which contained 271 mg/L HGA, showed similar results (Fig. 4b). The two peaks at 406 and 430 nm also decreased as the urine sample was diluted.

### Analysis by LC/TOF-MS spectrometry

**Figure 5:**
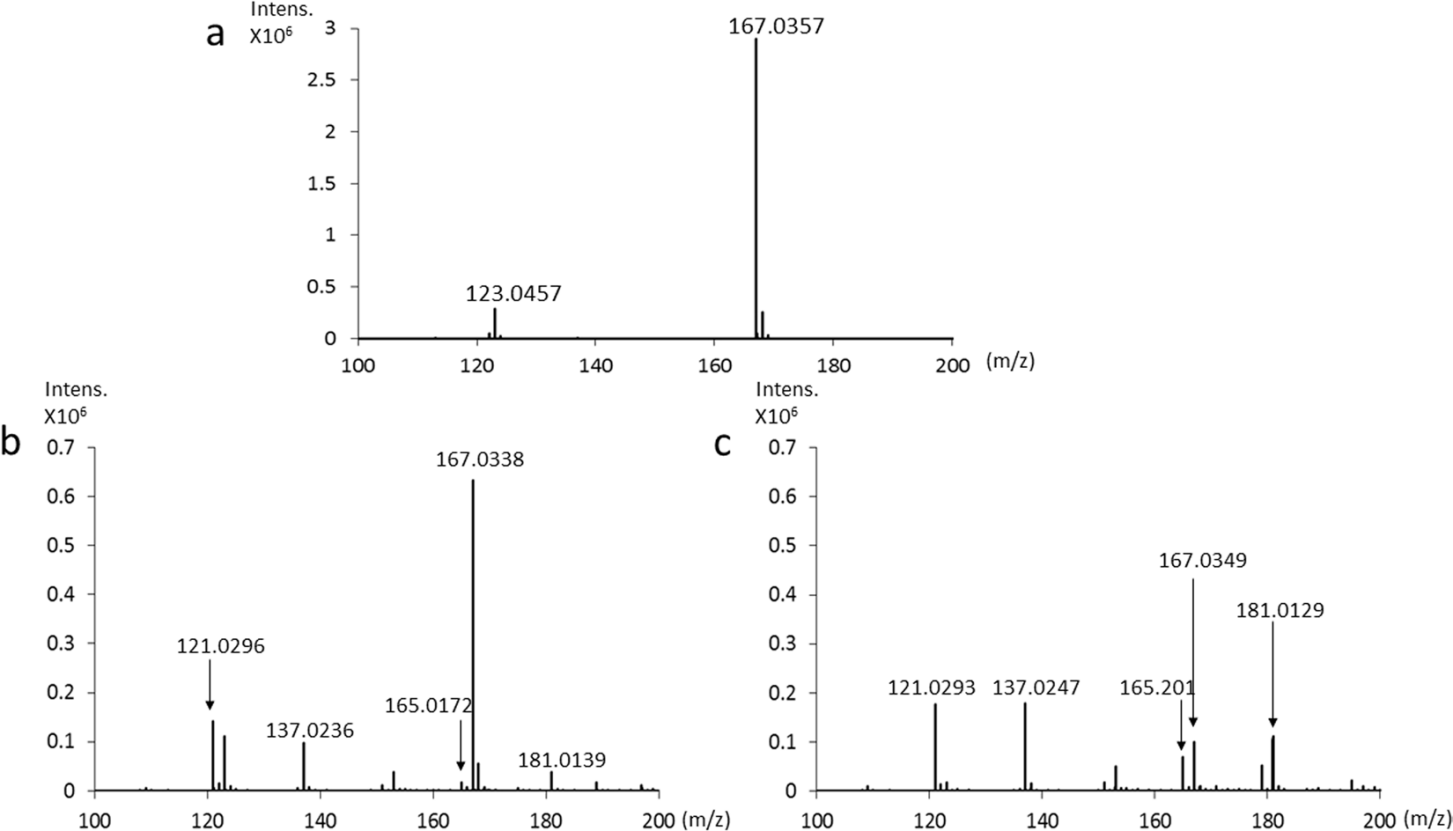
LC/TOF-MS spectra of 800 mg/L HGA. (**a**) MS spectrum of 800 mg/L HGA. (**b**) MS spectrum of 800 mg/L HGA after the addition of NaOH. (**c**) MS spectrum of 800 mg/L HGA after the addition of NaOH with NaOCl·5H_2_O.

We detected HGA and BQA using LC/TOF-MS. The HGA solution showed the molecular ion [HGA-H]^−^ at m/z 167 and a fragment at m/z 123 (Fig. 5a). The HGA solution after the addition of NaOH showed the molecular ion [BQA-H]^−^ at m/z 165 and a fragment at m/z 121 (Fig. 5b). In addition, metabolites of BQA were observed at m/z 181 (oxidation), which was 16 Da more than the [BQA-H]^−^ ion at m/z 165, and a fragment of the oxidant compound (m/z 181) was observed at m/z 137 (Fig. 5b). Similar ions were obtained for the HGA solution after the addition of NaOH with NaOCl·5H_2_O (Fig. 5c).

**Figure 6:**
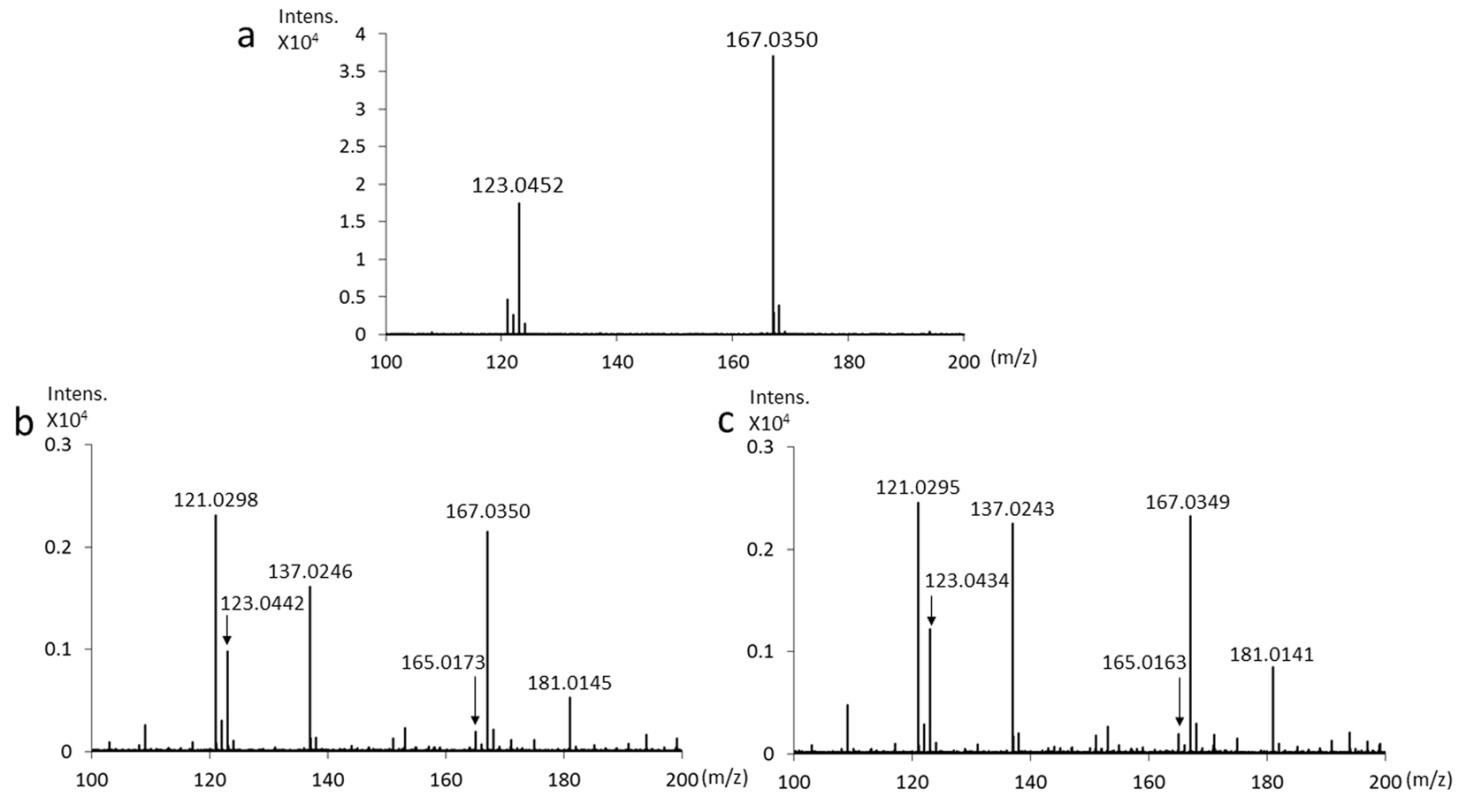
LC/TOF-MS spectra of alkaptonuric urine. (**a**) MS spectrum of alkaptonuric urine (HGA concentration of 271 mg/L). (**b**) MS spectrum of alkaptonuric urine after the addition of NaOH. (**c**) MS spectrum of alkaptonuric urine after the addition of NaOH with NaOCl·5H_2_O.

We also detected HGA and BQA in alkaptonuric urine. The urine sample from a patient with alkaptonuria showed the molecular ion [HGA-H]^−^ at m/z 167 and a fragment at m/z 123 (Fig. 6a). Alkaptonuric urine after the addition of NaOH showed the molecular ion [BQA-H]^−^ at m/z 165 and a fragment at m/z 121 (Fig. 6b). Moreover, the oxidant compound of BQA was observed at m/z 181 and its fragment was noted at m/z 137 (Fig. 6b). Similar ions were obtained for alkaptonuric urine after the addition of NaOH with NaOCl·5H_2_O (Fig. 6c).

### Analysis by NMR spectroscopy

**Figure 7:**
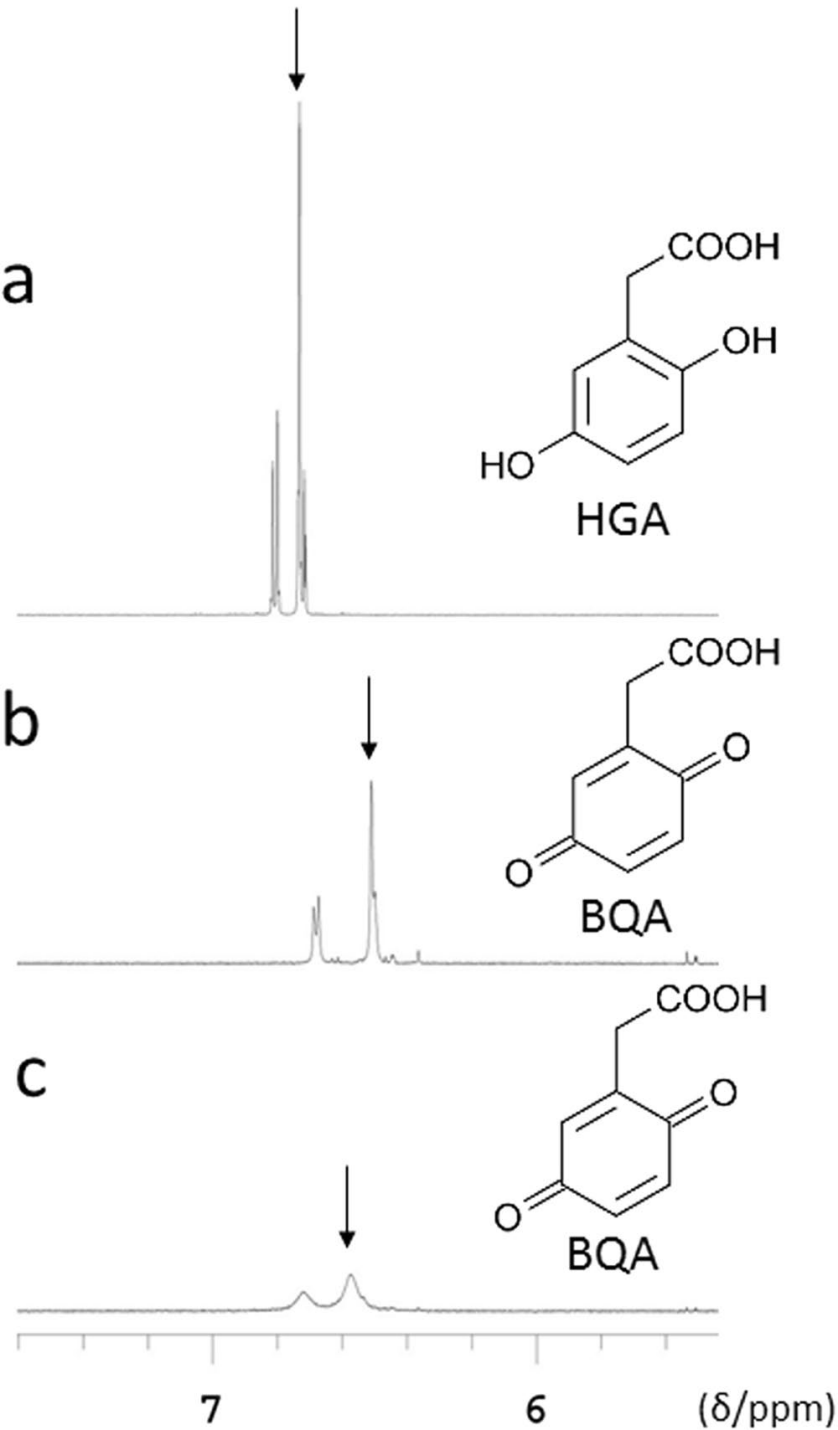
NMR spectra of 800 mg/L HGA. (**a**) Chemical shift in 800 mg/L HGA (D_2_O solution 1). The arrow indicates 6.73 ppm. (**b**) Chemical shift in 800 mg/L HGA after the addition of NaOH (D_2_O solution 2). The arrow indicates 6.50 ppm. (**c**) Chemical shift in 800 mg/L HGA after the addition of NaOH with NaOCl·5H_2_O (D_2_O solution 3). The arrow indicates 6.58 ppm.

We analyzed D_2_O solutions 1–3 (see the Methods) using NMR spectroscopy. The chemical shift in HGA, an aromatic proton adjacent to the acetic acid group of D_2_O solution 1, was a singlet at 6.73 ppm (Fig. 7a). The chemical shift in BQA, which was formed by the oxidation of HGA after the addition of NaOH (D_2_O solution 2), was a singlet at 6.50 ppm (Fig. 7b). The chemical shift in BQA incubated in HGA solution with NaOH with NaOCl·5H_2_O (D_2_O solution 3) was a singlet at 6.58 ppm, and a broadened singlet was enhanced by the addition of NaOCl·5H_2_O (Fig. 7c).

### Effects of interference substances

We examined how the interference substances, ascorbic acid (AA) and conjugated bilirubin, affected the absorption curve of HGA solution.

**Figure 8:**
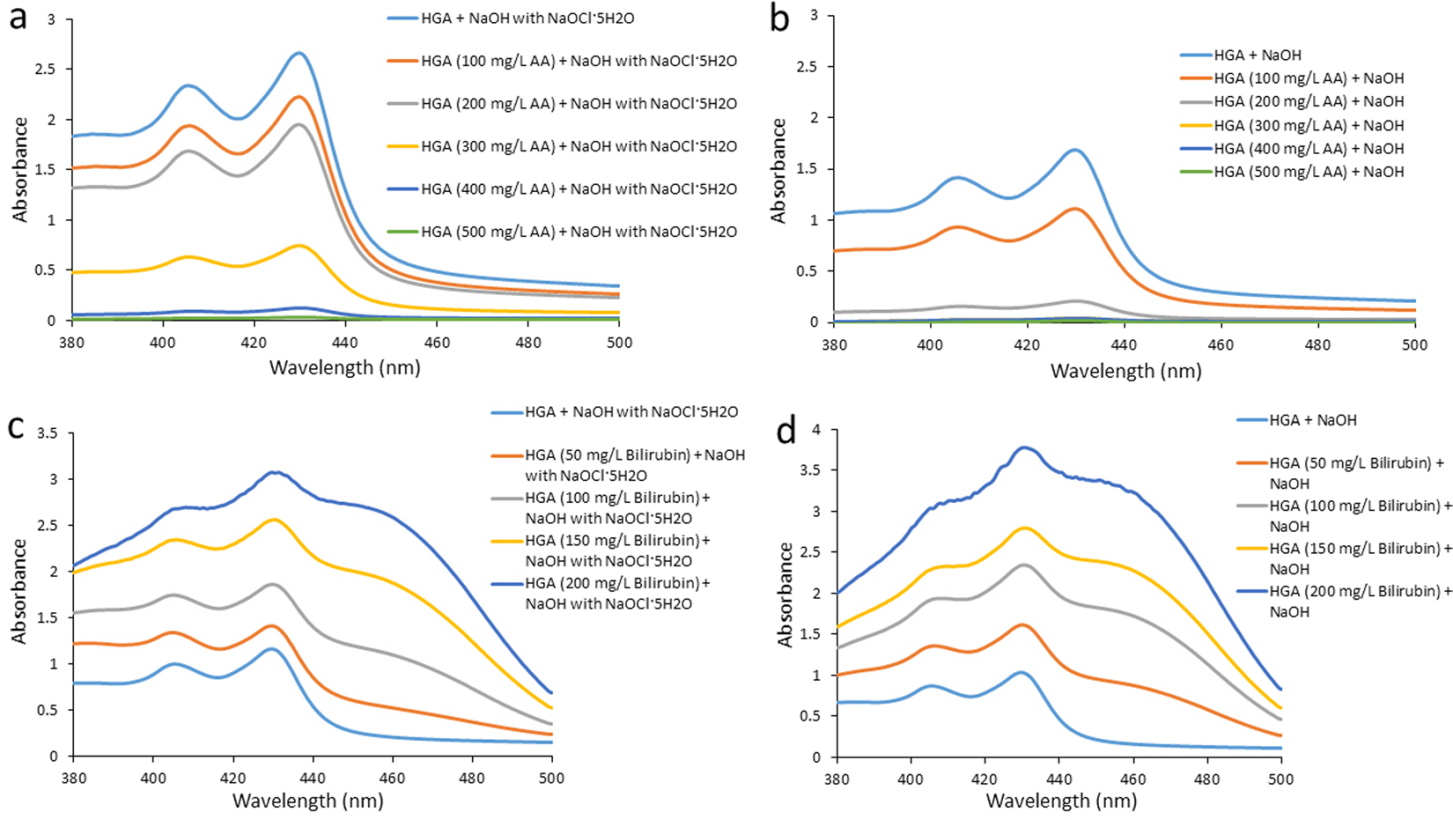
Effects of interference substances. NaOH with NaOCl·5H_2_O (**a**) or NaOH (**b**) was added to 800 mg/L HGA solutions containing several concentrations of AA (100, 200, 300, 400, and 500 mg/L). NaOH with NaOCl·5H_2_O (**c**) or NaOH (**d**) was added to 800 mg/L HGA solutions containing several concentrations of conjugated bilirubin (50, 100, 150, and 200 mg/L).

We added several concentrations of AA, an antioxidant, to HGA solution. The spectrum of HGA solution treated with more than 400 mg/L AA showed no peaks at 406 and 430 nm following the addition of NaOH with NaOCl·5H_2_O (Fig. 8a). Furthermore, the spectrum of HGA solution treated with more than 200 mg/L AA showed no peaks at 406 and 430 nm following the addition of NaOH (Fig. 8b).

We then added several concentrations of conjugated bilirubin, a water-soluble bilirubin, to HGA solution. The absorbance spectra of HGA solution after the addition of NaOH with NaOCl·5H_2_O increased in a dose-dependent manner of conjugated bilirubin (Fig. 8c). The peak at 406 nm of HGA solution containing 200 mg/L conjugated bilirubin after the addition of NaOH with NaOCl·5H_2_O almost disappeared. Similar results were obtained for HGA solution after the addition of NaOH (Fig. 8d).

## Discussion

In the present study, we accelerated the oxidation of HGA to BQA incubated with NaOH with NaOCl·5H_2_O and observed the oxidation reaction after alkalization by LC/TOF-MS and NMR spectrometry. Our results revealed that alkaptonuric urine and HGA solution became a darker brown following the addition of NaOH with NaOCl·5H_2_O than after the addition of NaOH. Furthermore, regarding the urine sample and HGA solution, absorbance values at 406 and 430 nm were higher following the addition of NaOH with NaOCl·5H_2_O than after the addition of NaOH. A comparison with analyses of absorption spectra in the UV region and LC/TOF-MS and NMR spectrometry revealed that absorption spectra in the visible region more closely reflected the color change after alkalization. These results may contribute to the development of a new rapid method for assessing the oxidation of HGA to BQA in urine by measuring absorption spectra in the visible region after an incubation with NaOH with NaOCl·5H_2_O.

Previous studies reported that the HGA solution shows a characteristic peak at 290 nm; a second peak gradually begins to appear at 250 nm with the oxidation of HGA to BQA^3,4,11^. Therefore, a number of spectrophotometric studies have been conducted in the UV region of alkaptonuric urine and HGA solution. However, when measuring absorption spectra in the UV region, samples need to be diluted dozens to several hundred times, and absorption peaks are not stable. Based on these findings, we developed a rapid identification test for alkaptonuria after alkalization based on the measurement of absorption spectra in the visible region^10^. In measurements of absorption spectra in the visible region, samples are used without dilution or following an approximately 2-fold dilution, and absorption peaks are stable. As shown in Figs 1b, 2b and 3, two characteristic peaks at 406 and 430 nm after alkalization disappeared at the time of the measurement of absorption spectra in the UV region. Due to differences in dilution degrees, the characteristic peaks of alkaptonuric urine or HGA solution in the UV and visible regions after alkalization for 3 min were not measureable at the same time (Figs 1b, 2b and 3). Moreover, the intensity of the dark brown color and absorbance values at 406 and 430 nm of HGA solution were significantly higher following the addition of NaOH with NaOCl·5H_2_O than with the addition of NaOH (Fig. 2b,c). However, a significant difference was not observed between the absorbance values at 250 nm after the addition of NaOH with NaOCl·5H_2_O and with the addition of NaOH (Fig. 3a). Based on these results, the measurement of the absorption curve in the visible region sensitively reflects color changes in HGA after alkalization.

The urine sample from the patient with alkaptonuria and HGA solution incubated with NaOCl·5H_2_O did not show a color change (Figs 1a and 2a). This was attributed to the pH of alkaptonuric urine and HGA solution not being alkaline, and the color of acidic alkaptonuric urine (pH 6.0) did not change at room temperature for a few days (data not shown). The oxidation of HGA to BQA is known to be accelerated by NaOH. Oxygen consumption by HGA increases at an alkaline pH^12,13^. Therefore, the addition of a combination of alkaline solution and NaOCl·5H_2_O effectively accelerated the oxidation of HGA to BQA.

We developed a method for the rapid oxidation of HGA incubated with NaOH with NaOCl·5H_2_O and this method detected HGA from 180 to 400 mg/L (Fig. 4a); however, HGA solutions containing more than 400 mg/L require dilution prior to absorbance measurements due to the darker brown color following the addition of NaOH with NaOCl·5H_2_O (data not shown). A previous study reported that urine from a patient with alkaptonuria contained excessive amounts of HGA ranging between 1830 and 3502 mg/L, whereas the urinary HGA concentrations of healthy children were less than 12.26 mg/L^14^. As described above, the urinary HGA levels of patients with alkaptonuria were dozens to several hundred times higher than those of healthy subjects. In the present study, we analyzed urine from a patient with alkaptonuria in which the HGA concentration was measured as 271 mg/L by liquid chromatography-tandem mass spectrometry (LC-MS/MS). This alkaptonuric urine showed peaks at 406 and 430 nm (Fig. 1b), and these two peaks decreased as the urine sample was diluted by 4/10 (108 mg/L) (Fig. 4b). Therefore, alkaptonuria was detected by the addition of NaOH with NaOCl·5H_2_O to urine.

We previously measured the absorption spectra of urine samples from phenylketonuria patients and solutions of 2-hydroxyphenylacetic acid after the addition of NaOH in order to examine the specificity of the two peaks at 406 and 430 nm after alkalization, and confirmed that these two peaks after the addition of NaOH were specific for HGA and useful for the diagnosis of alkaptonuria^10^. We measured the absorption spectra of urine samples from phenylketonuria patients, solutions of 2-hydroxyphenylacetic acid, and solutions of phenylalanine after the addition of NaOH with NaOCl·5H_2_O. The results of these measurements showed that the absorption curve of the urine samples, 2-hydroxyphenylacetic acid, and phenylalanine did not have any peak at 406 or 430 nm (data not shown). These results suggest that the method for the rapid oxidation of HGA incubated with NaOH with NaOCl·5H_2_O is also useful for the diagnosis of alkaptonuria.

Analyses of HGA in urine using NMR spectrometry and HPLC have been reported^8,9^. However, the oxidation reaction of HGA to BQA after alkalization has not been examined by LC/TOF-MS and NMR spectrometry. After the addition of NaOH to HGA, the LC/TOF-MS analysis showed that the ion intensity of HGA at m/z 167 decreased, that of BQA at m/z 165 increased, and the oxidant compound of BQA at m/z 181 also formed (Fig. 5a,b). Furthermore, the LC/TOF-MS analysis showed that the ion intensity of HGA at m/z 167 was lower, that of BQA at m/z 165 was higher, and that of the oxidant compound of BQA at m/z 181 was higher following the addition of NaOH with NaOCl·5H_2_O than after the addition of NaOH (Fig. 5b,c). The LC/TOF-MS analysis of alkaptonuric urine showed similar molecular ions (Fig. 6). The oxidant compound of BQA at m/z 181 and its fragment at m/z 137 were higher following the addition of NaOH with NaOCl·5H_2_O than after the addition of NaOH (Fig. 6b,c). These results of the LC/TOF-MS analysis indicate that NaOCl·5H_2_O significantly accelerates the oxidation of HGA to BQA. As shown in Fig. 7, NMR spectroscopy revealed that the chemical shift in BQA formed by the oxidation of HGA indicated a broadened singlet, and this singlet was enhanced by the addition of NaOCl·5H_2_O because a decrease in the solubility of BQA for the solvent accompanied by the oxidation of HGA to BQA resulted in a broadened singlet of BQA. As BQA solubility for the solvent decreased, the ion intensity of BQA at m/z 165 was lower than HGA at m/z 167 in the analysis by LC/TOF-MS spectrometry (Figs 5b,c and 6b,c). Based on the results of analyses by LC/TOF-MS and NMR spectrometry, the oxidation of HGA to BQA was accelerated by the addition of HGA to NaOH, and was further accelerated by the addition of NaOH with NaOCl·5H_2_O. However, due to decreases in the solubility of BQA for the solvent accompanied by the oxidation of HGA to BQA, the detection sensitivities of BQA analyzed by LC/TOF-MS and NMR spectroscopy are not high.

We previously reported that alkaptonuric urine and HGA solution treated with AA showed no peaks at 406 or 430 nm following the addition of NaOH^10^. In order to confirm the effects of an antioxidant, we investigated whether the two peaks at 406 and 430 nm were present after the addition of NaOH with NaOCl·5H_2_O (Fig. 8a). The spectra of HGA solution containing more than 400 mg/L AA showed no peaks at 406 or 430 nm following the addition of NaOH with NaOCl·5H_2_O (Fig. 8a), and HGA solution containing more than 200 mg/L AA showed no peaks following the addition of NaOH (Fig. 8b). Therefore, in spite of the addition of NaOH with the oxidant NaOCl·5H_2_O to urine, alkaptonuric patients receiving high doses of AA may yield false negative results. We also investigated the effects of conjugated bilirubin. The absorbance of HGA solution at 430 and 406 nm increased in a dose-dependent manner of conjugated bilirubin (Fig. 8c,d). These results indicate that our spectrometric method for the detection of HGA is suitable for a qualitative rather than quantitative analysis because the absorbance values of two peaks at 430 and 406 nm were increased by the effects of conjugated bilirubin and other pigments in urine.

In summary, we accelerated the oxidation of HGA to BQA after alkalization by adding NaOH with NaOCl·5H_2_O, and BQA levels were markedly higher following the addition of NaOH with NaOCl·5H_2_O to HGA than with the addition of NaOH. Moreover, absorbance measurements in the visible region are useful for observing this color change because the oxidation of HGA to BQA is more sensitive than absorbance measurements in the UV region, LC/TOF-MS, and NMR spectroscopy. This simple, quick, and highly sensitive method may be suitable for detecting BQA in urine and diagnosing alkaptonuria.

## Methods

### Patient

One alkaptonuria patient was diagnosed at the University of Tokyo Hospital. The clinical diagnosis of alkaptonuria was reached based on the detection of the dark brown pigmentation of cartilage and connective tissues during surgery for osteoarthritis of the knee. The diagnosis of alkaptonuria was confirmed by the detection of HGA in a urine sample using LC-MS/MS and a large quantity of HGA (271 mg/L) in urine. Urine was collected from the patient. Written informed consent from a patient was obtained for the use of the urine samples. This study was conducted with the approval of the Institutional Research Ethics Committee of the Faculty of Medicine, the University of Tokyo (Approval no. 3333-34) and Ehime Prefectural University of Health Sciences (Approval no. 16-024) and performed in accordance with the Ethical Guidelines for Medical and Health Research Involving Human Subjects.

### Reagents

Sodium hydroxide (NaOH) was purchased from Wako Pure Chemical Industries, Ltd. (Osaka, Japan). HGA was purchased from Tokyo Chemical Industry Co., Ltd. (Tokyo, Japan). NaOCl·5H_2_O was obtained from Kaneka Co., Ltd. (Osaka, Japan). AA and conjugated bilirubin were available from Interference Check.A Plus purchased from Sysmex Co., Ltd. (Kobe, Japan).

### Transient spectrum measurement

Sample solutions were measured using a model U-2900 spectrophotometer (Hitachi High-Technology Co., Ltd., Tokyo, Japan) with microcells with a 10-mm path length. Regarding alkalization, 10 μL of 1 M NaOH or 1 M NaOH with NaOCl·5H_2_O (2 wt%) was added to 0.8 mL of alkaptonuric urine (HGA concentration of 271 mg/L), alkaptonuric urine samples diluted with distilled water (10/10, 9/10, 8/10, 7/10, 6/10, 5/10, 4/10, 3/10, 2/10, and 1/10 dilution), or HGA in distilled water (800, 400, 300, 200, 190, 180, 170, 160, 150, 140, 130, 120, 110, and 100 mg/L). Sample solutions were incubated at room temperature for 3 min. In each absorption measurement in the visible region, 800 mg/L HGA was diluted 2 times with distilled water after an incubation with NaOH or 1 M NaOH with NaOCl·5H_2_O, and in the UV region, 800 mg/L HGA and alkaptonuric urine were diluted 40 times with distilled water after an incubation with NaOH or 1 M NaOH with NaOCl·5H_2_O. All measurements were performed with a 1-nm bandwidth at a scan speed of 100 nm/min, and distilled water was set as the blank.

### LC/TOF-MS spectrometry

Flow injection LC/TOF-MS analyses were performed on a Shimadzu Corporation Nexera X2 UHPLC System and Bruker Daltonics maXis 4 G. Regarding alkalization, 10 μL of 1 M NaOH or 1 M NaOH with NaOCl·5H_2_O (2 wt%) was added to 0.8 mL of 800 mg/L HGA in distilled water or alkaptonuric urine. Sample solutions were incubated at room temperature for 3 min and introduced directly to LC/TOF-MS without passing through the column. The mobile phase was 50% methanol aqueous solution and the flow rate was 0.2 mL/min. The ESI capillary voltage was set at 3000 V, fragmentor voltage at 150 V, gas temperature at 200 °C, gas flow at 2 mL/min, and nebulizer pressure at 1 bar. Mass spectra (m/z 50–1500) were acquired in the negative-ion mode.

### NMR spectroscopy

A D_2_O solution of 800 mg/L HGA was prepared (D_2_O solution 1). Ten microliters of 1 M NaOH solution was added to 0.8 mL of this solution (D_2_O solution 2). Ten microliters of 1 M NaOH solution with NaOCl·5H_2_O (2 wt%) was added to 0.8 mL of D_2_O solution of 800 mg/L HGA (D_2_O solution 3). Three types of D_2_O solutions were prepared and subjected to NMR measurements. All NMR data were recorded on a Varian VNMRS 600 spectrometer, operating at 599.936 MHz for protons. NMR spectra were acquired at 293 K using 5-mm NMR tubes. 4, 4-Dimethyl-4-silapentane-1-sulfonic acid (DSS) was used as an internal standard for the chemical-shift calibration. ^1^H NMR spectra were recorded using the standard pulse sequence and setting the repetition time and number of scans to 13.4 s and from 16 to 64 scans, respectively.

### Interference assay

In order to examine the effects of AA, 10 μL of 1 M NaOH or 1 M NaOH with NaOCl·5H_2_O (2 wt%) was added to 0.8 mL of 800 mg/L HGA containing several concentrations of AA (100, 200, 300, 400, and 500 mg/L). Sample solutions were incubated at room temperature for 3 min. In each absorption measurement in the visible region, samples was diluted 2 times with distilled water after an incubation with NaOH or 1 M NaOH with NaOCl·5H_2_O.

In order to examine the effects of conjugated bilirubin, 10 μL of 1 M NaOH or 1 M NaOH with NaOCl·5H_2_O (2 wt%) was added to 0.8 mL of 800 mg/L HGA containing several concentrations of conjugated bilirubin (50, 100, 150, and 200 mg/L). Sample solutions were incubated at room temperature for 3 min. In each absorption measurement in the visible region, samples was diluted 4 times with distilled water after an incubation with NaOH or 1 M NaOH with NaOCl·5H_2_O.

### Statistical analysis

The significance of differences between two groups was assessed using the 2-sample *t*-test because the normality and equality of the variance were validated in various groups. All data were expressed as the mean ± SD. A value of *P* < 0.05 was considered to be significant. All analyses were performed using StatFlex software (version 6.0; Artec Inc., Osaka, Japan).
